# Crystal structure of a family VIII β‐lactamase fold hydrolase reveals the molecular mechanism for its broad substrate scope

**DOI:** 10.1111/febs.16554

**Published:** 2022-06-27

**Authors:** Isabel Cea‐Rama, Cristina Coscolín, Jose L. Gonzalez‐Alfonso, Jog Raj, Marko Vasiljević, Francisco J. Plou, Manuel Ferrer, Julia Sanz‐Aparicio

**Affiliations:** ^1^ IQFR, CSIC Madrid Spain; ^2^ ICP, CSIC Madrid Spain; ^3^ PATENT CO, DOO Mišićevo Serbia

**Keywords:** esterase, metagenome, promiscuity, α/β‐fold hydrolase, β‐lactamase

## Abstract

Family VIII esterases present similarities to class C β‐lactamases, which show nucleophilic serines located at the S‐X‐X‐K motif instead of the G‐X‐S‐X‐G or G‐D‐S‐(L) motif shown by other carboxylesterase families. Here, we report the crystal structure of a novel family VIII (subfamily VIII. I) esterase (EH_7_; denaturing temperature, 52.6 ± 0.3 °C; pH optimum 7.0–9.0) to deepen its broad substrate range. Indeed, the analysis of the substrate specificity revealed its capacity to hydrolyse nitrocefin as a model chromogenic cephalosporin substrate (40.4 ± 11.4 units·g^−1^), and a large battery of 66 structurally different esters (up to 1730 min^−1^), including bis(2‐hydroxyethyl)‐terephthalate (241.7 ± 8.5 units·g^−1^) and the mycotoxin T‐2 (1220 ± 52 units·g^−1^). It also showed acyltransferase activity through the synthesis of benzyl 3‐oxobutanoate (40.4 ± 11.4 units·g^−1^) from benzyl alcohol and vinyl acetoacetate. Such a broad substrate scope is rare among family VIII esterases and lipolytic enzymes. Structural analyses of free and substrate‐bound forms of this homooctamer esterase suggest that EH_7_ presents a more opened and exposed S1 site having no steric hindrance for the entrance of substrates to the active site, more flexible R1, R2 and R3 regions allowing for the binding of a wide spectrum of substrates into the active site, and small residues in the conserved motif Y‐X‐X containing the catalytic Tyr enabling the entrance of large substrates. These unique structural elements in combination with docking experiments allowed us to gain valuable insights into the substrate specificity of this esterase and possible others belonging to family VIII.

## Graphical abstract

We present a comprehensive biochemical and structural study of a family VIII ester hydrolase with a broad and unusual substrate profile, including acyltransferase activities. A wide catalytic cavity accepts an ample set of different esters, some being relevant to plastic and mycotoxin degradation. Depiction of detailed interaction with substrates unravels the plasticity of binding modes and provides valuable insights into the substrate specificity of this esterase and others within family VIII.

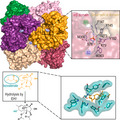

**Abbreviations**
BHET: bis(2‐hydroxyethyl)‐terephthalate
BR: Britton–Robinson
EH: Ester Hydrolase
EPPS: 4‐(2‐hydroxyethyl)‐1‐piperazinepropanesulfonic acid
HEPES: N‐(2‐hydroxyethyl)piperazine‐N′‐(2‐ethanesulfonic acid)
M‐4NHP: methyl 4‐nitrophenyl hexylphosphonate
MHET: mono‐(2‐hydroxyethyl)‐terephthalic acid
O‐4NHP: octyl 4‐nitrophenyl hexylphosphonate
PET: polyethylene terephthalate
*p*‐NP: p‐nitrophenyl
TA: terephthalic acid

## Introduction

Esterases and lipases of the α/β‐hydrolase fold family may support multiple conversions of interest in a modern circular bioeconomy [1]. Although esterases and lipases differ in terms of sequence identity, structural motifs and substrate specificity, particularly the preference for substrates with short or long lengths in the acyl region, they commonly share a number of interesting features and advantages compared with other biocatalysts. In particular, these include their broad abundance in any organism, easy identification, production and purification, stability, and, most importantly, the possibility of supporting enantioselective conversions [1]. Overall, approximately three hundred thousand protein sequences have been catalogued and integrated into at least 35 families and 11 true lipase subfamilies [2, 3]. Although this classification is based on sequence, advances in molecular biology, high‐throughput biochemical assays and structural actions (approximately 1600 protein structures available according to the lipase engineering database [3]) have revealed a variety of architectures and biochemical features. We know that mobile interfacial‐activated lids, immobile cap domains, and the presence of conserved motifs and oxyanion hole signatures, together with specific key amino acids close to or in the proximity of the active sites, determine the entrance to, or positioning of substrates in, the esterase and lipase's active site [3]. The accumulated experimental information on the substrate recognition of close to 145 esterases has further demonstrated that those with a broader substrate range are characterized by active sites with higher effective volume [4] and lower structural flexibility [5]. In addition, sequence‐substrate spectrum relationships have revealed that substrate promiscuity is a characteristic most prevalent in members of family IV, also called the hormone‐sensitive lipase (HSL) family [4]. Recent structural analysis has suggested that the substrate promiscuity that was frequently observed for this esterase family is due to a substrate prerecognition mechanism by hydrophobic patches of the cap domains [6].

Noticeably, among the set of approx. 145 esterases for which the substrate promiscuity has been investigated at large [4, 5], a family VIII serine β‐lactamase, named EH_7_ (EH, abbreviation of ester hydrolase), also showed a substrate spectrum as wide as that of members of family IV (it was ranked as the seventh with the highest substrate specificity). Family VIII is within the superfamily of α/β‐folding hydrolases. Members of this family are most similar to class C, one of the four groups into which β‐lactamases are classified on the basis of sequence similarities and conserved motifs [7]. β‐Lactamases are characterized by three conserved structural motifs. The first motif is the S‐X‐X‐K motif, where the two catalytic residues Ser and Lys are located. The second conserved motif is the S‐D‐N loop that shapes one of the walls of the catalytic cavity. Class C β‐lactamases and family VIII include Tyr, which is completing the catalytic triad. Finally, the third conserved motif, which is part of the other wall of the catalytic cavity, is the K‐T‐G box. Family VIII is further subdivided into 3 subfamilies, VIII.1, VIII.2 and VIII.3, according to the conservation or absence of the previous motifs [8] [9]. Subfamily VIII.2 has a fourth conserved motif, G‐X‐S‐X‐G, that is common to family IV, in which the nucleophilic serine is included [10] (Table 1).

**Table 1:** Conserved motifs among class C β‐lactamases and the three class VIII subfamilies.

	**S**‐X‐X‐**K**	G‐X‐S‐X‐G	S‐D‐N	K‐T‐G
Class C β‐lactamases	Yes	No	**Y**‐A‐N	Yes
Subfamily VIII.1	Yes	No	**Y**‐X‐X	W‐X‐G
Subfamily VIII.2	Yes	Yes	**Y**‐X‐X	W‐X‐G
Subfamily VIII.3	Yes	No	**Y**‐X‐X	H‐X‐X

The catalytic Tyr is in bold.

Structurally, members of family VIII present an insertion domain and an α/β domain, with the active site located at the interface between the two domains and surrounded by three variable regions of the protein, two being within the α/β domain and one at the insertion domain. One of the structural reasons associated with β‐lactamase activity is due to one of these regions, as it may prevent the hydrolysis of β‐lactam substrates due to steric hindrance [11].

In relation to their substrate spectra, members of family VIII are capable of hydrolysing short‐chain esters [12], but their main interest lies in their ability to hydrolyse β‐lactam antibiotics [13, 14] and, in some cases, phthalate mono‐ and diesters [10]. Although the substrate promiscuity of β‐lactamases with regard to the ability to degrade antibiotics has been studied in detail [11, 13, 14], little is known about the mechanisms by which some of the family VIII esterases similar to β‐lactamases are also able to hydrolyse a wide variety of esters, a capacity exemplified by the enzyme EH_7_. This enzyme was previously reported to have a promiscuous substrate character (by the number of hydrolysed substrates) related to the high effective volume of its active site and its reduced structural flexibility compared with other esterases [4, 5]. To further elucidate the molecular mechanism determining the wide and unusual substrate range of this esterase, we report the extensive biochemical characterization of the family VIII esterase EH_7_ and solved its crystal structure in the apo form and with two bound substrate analogues.

## Results and Discussion

### Biochemical and substrate specificity characteristics of EH_7_

EH_7_ (GeneBank acc. Nr. KY483644; molecular mass, 45 398 Da; isoelectric point, 5.52) is an esterase isolated from the metagenomic DNA of microbial communities inhabiting the chronically polluted seashore area of Milazzo Harbour in Sicily [4]. TBLASTX analysis revealed compositional similarities between the DNA fragment containing the gene for EH_7_ and genomic sequences of a bacterium from the genus *Pseudomonas* [4]. This enzyme belongs to family VIII (subfamily VIII. of lipolytic enzymes that present similarities to class C β‐lactamases or AmpC), which show a nucleophilic serine located at the S‐X‐X‐K motif instead of the G‐X‐S‐X‐G motif shown by other carboxylesterase families [3]. Most enzymes within this family lack activity on β‐lactamic substrates, although exceptions have been reported [11].

**Fig. 1:**
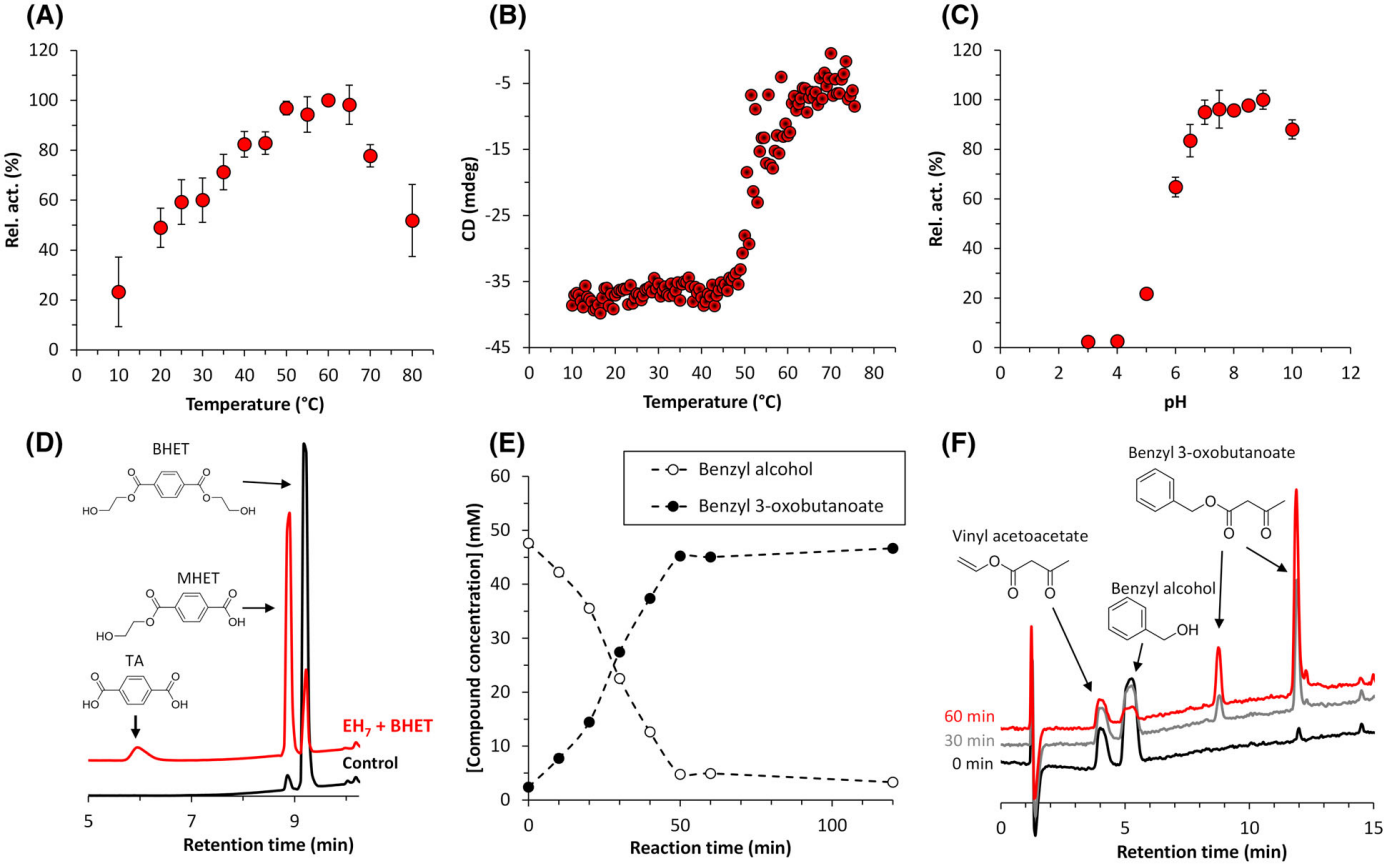
Biochemical characterization of EH_7_. (A) Temperature profile. (B) The thermal denaturation curve of EH_7_ at pH 7.0, as determined by circular dichroism measuring the ellipticity changes at 220 nm obtained at different temperatures at a rate of 0.5 °C per min (conditions as in [5]). (C) The pH profile. In A and C, the maximal activity was defined as 100%, and relative activity is shown as percentage of maximal activity (mean ± SD of triplicates) determined under conditions described in Materials and methods. (D) HPLC chromatogram representing the degradation products when BHET was incubated (red) or not (black) with EH_7_. (E) Concentration of the substrate and reaction product representing acyltransferase activity, as determined by HPLC. (F) HPLC chromatogram representing the synthesis of benzyl 3‐oxobutanoate. Note: Under our analysis conditions, the product benzyl 3‐oxobutanoate eluted as two peaks, which correspond to those observed in the pure compound. The figure was created using Excel 2019.

**Fig. 2:**
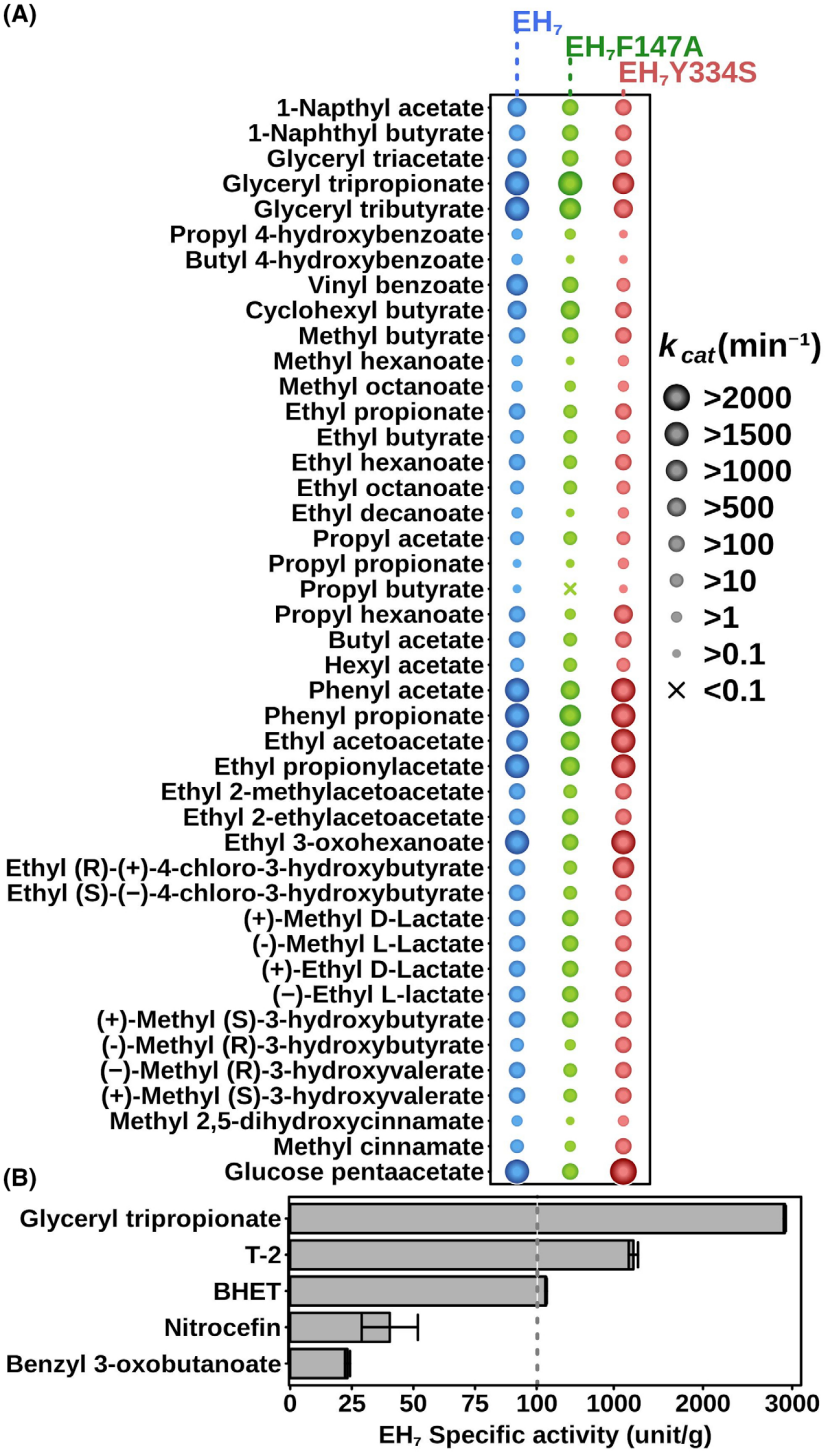
Substrate specificity. (A) The *k*_cat_ (min^−1^) values (mean values with deviations of triplicates being < 5%) of the EH_7_, EH_7F147A_ and EH_7Y334S_ variants measured for 43 carboxylic esters out of the 64 hydrolysed by EH_7_ [5]. Note that the data for EH_7_ were obtained from [5], where as those for the mutants are herein reported for the first time. (B) Specific activity (unit·g^−1^; mean ± SD of triplicates) for 5 model substrates. Activity was determined under conditions described in Materials and methods.

EH_7_ showed maximal activity at 50 °C (Fig. 1A). The fact that its denaturing temperature was 52.55 ± 0.31 °C, as determined by circular dichroism analysis (Fig. 1B), suggests that the enzyme does not denature at the optimal temperature during our assay conditions, although under other assay conditions (*e.g*. assay time), the optimum temperature plot may differ. Its optimal pH for activity is 7.0–9.0 (Fig. 1C). As reported previously [4, 5], using a pH‐indicator assay (pH 8.0, 30 °C), the enzyme was capable of hydrolysing 64 structurally different esters according to the Tanimoto‐Combo similarity score, with *k*_cat_ values ranging from 1683 (for glyceryl tripropionate) to 0.16 (for methyl ferulate) min^−1^ (Fig. 2A). In this study, we further tested the possibility that the enzyme hydrolyses other substrates (Fig. 2B).

First, we found that EH_7_ efficiently hydrolysed the mycotoxin T‐2, with a specific activity determined using a pH‐indicator assay at pH 8.0 and 30 °C of 1220 ± 52 units·g^−1^ (Fig. 2B); this rate is similar to that for the best ester substrate, glyceryl tripripionate (2918 ± 12 units·g^−1^). T‐2 reaction products were analysed by electrospray ionization mass spectrometry (Fig. S1). The reaction with toxin ionized as (M + NH_4_)^+^ with an *m*/*z* of 484 and as (M + Na)^+^ with an *m*/*z* of 489. On the other hand, when T‐2 was incubated with EH_7_, a product ionized as (M + NH_4_)^+^ with an *m*/*z* of 400 and as (M + Na)^+^ with an *m*/*z* of 405 Da and with 84 units less than the T‐2 substrate molecule. This result demonstrates that EH_7_ degrades T‐2 by removing the isovalerate ester group, thus producing neosolaniol. We would like to highlight that mycotoxins can be degraded by enzymes and microbes, but enzymes capable of degrading mycotoxins such as T‐2 toxin are rare. Indeed, only hydrolases degrading T‐2 to the similarly toxic intermediate HT‐2 are known [15], and a few examples of microbes degrading T‐2 to neosolaniol have been reported, although the enzymes responsible for its degradation have not been identified [15, 16, 17].

Second, by using a pH‐indicator assay (pH 8.0, 30 °C), we also found that EH_7_ efficiently hydrolysed bis(2‐hydroxyethyl)‐terephthalate (BHET), with a specific activity of 241.7 ± 8.5 units·g^−1^ (Fig. 2B), an intermediate in the degradation of polyethylene terephthalate (PET) [18]; high‐performance liquid chromatography (HPLC) analysis (Fig. 1D), performed as described [19], confirmed the hydrolysis of BHET to mono‐(2‐hydroxyethyl)‐terephthalic acid (MHET) and terephthalic acid (TA). Indeed, when using 4 mm BHET, we found a total degradation of 82% (8% to TA and 74% to MHET). It is to highlight that PETases degrade BHET to MHET without producing TA; rather, MHET is degraded by MHETases that in contrast to PETases are highly efficient for the hydrolysis of MHET to TA (up to *ca*. 25 s^−1^) but degrade BHET at a very low rate (*ca*. 0.0011 s^−1^) [20]. Therefore, the capacity of EH_7_ to efficiently hydrolyse BHET to MHET and TA is a unique feature of this enzyme not only among family VIII β‐lactamases but also among PET‐degrading hydrolases.

Third, nitrocefin was also accepted as a substrate (*K*_M_, 124.76 ± 6.59 μm), albeit at a low rate (40.4 ± 11.4 units·g^−1^, at pH 8.0, 30 °C; Fig. 2B). Finally, the enzyme also catalysed the synthesis of benzyl 3‐oxobutanoate when incubating benzyl alcohol with a fourfold excess of vinyl acetoacetate (Fig. 1E,F), with a specific activity of 23.26 ± 1.02 units·g^−1^ (pH 8.0, 30 °C) (Fig. 2B), and thus has acyltransferase activity.

### EH_7_ adopts the β‐lactamase fold

The crystal structure of wild‐type EH_7_ was obtained at 2.25 Å resolution, with the P4_3_22 space group and four crystallography‐independent molecules in the asymmetric unit. Molecular replacement was performed using EstU1 as a template (PDB code 4IVK) [11], and the final model was refined to a crystallographic R‐factor of 0.1874 and R‐free of 0.2270. The structures of the two EH_7_ complexes were obtained by soaking the native crystals in a solution containing either methyl 4‐nitrophenyl hexylphosphonate (EH_7_‐M‐4NHP) or octyl 4‐nitrophenyl hexylphosphonate (EH_7_‐O‐4NHP) were also solved at resolutions of 2.65 and 2.92 Å, respectively. Soaking of the crystals increases the volume of their unit cell, which belongs to the P4_3_2_1_2 space group and contains eight molecules in the asymmetric unit, including one ligand bound per catalytic site. The crystal structures of these complexes were solved using the coordinates of wild‐type EH_7_, and the final models were refined to crystallographic R factors of 0.1960 and 0.1835 and R‐free values of 0.2165 and 0.2210, respectively.

**Fig. 3:**
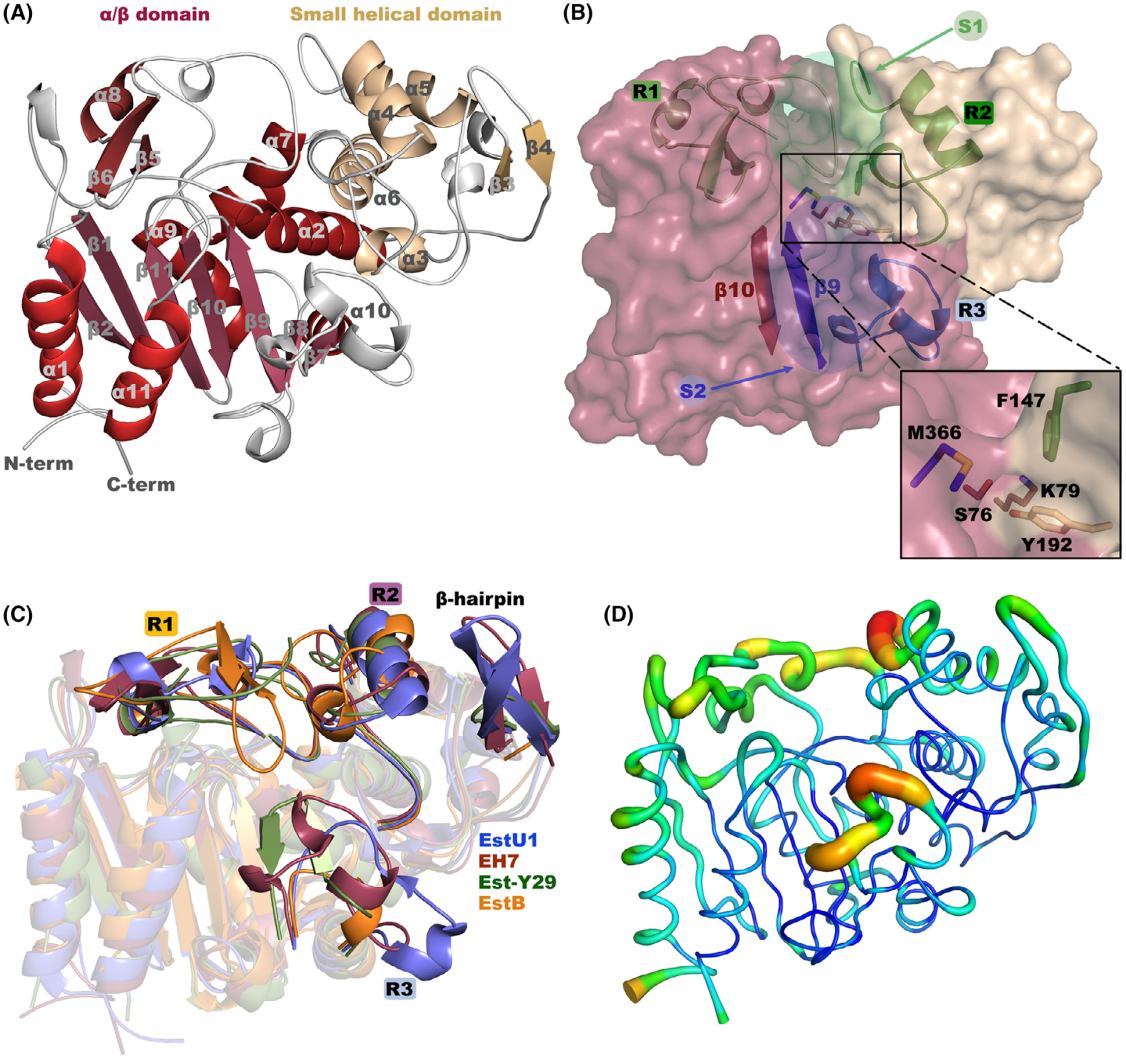
EH_7_ crystal structure. (A) General folding showing the α/β domain (red) and the small helical domain (wheat). (B) Depiction of the EH_7_ surface showing the sites S1 (outlined by R1 and R2) and S2 (defined by β9 and R3). Inset: Magnified image of the catalytic pocket. Residues of the catalytic triad are Ser76, Lys79 (located at the α/β domain, raspberry) and Tyr192 (located at the small helical domain, wheat). Residues Met366 (located at the β9‐ β10 loop, blue) and Phe147 (located in the R2 region, green) form the tunnel, giving access to the catalytic pocket. (C) Superimposition of the EH_7_ monomer (prune) and its homologues EstU1 (slate, PDB code 4IVI), Est‐Y29 (green, PDB code 4P6B) and EstB (orange, PDB code 1CI8). The R1 and R2 regions present the largest differences affecting access to the S1 site. (D) EH_7_ B‐factor values, from low (blue) to red (high). Most flexible regions are R2 and R3. All these figures were depicted using the software pymol (PyMOL Molecular Graphics System, Schrödinger LLC).

Similar to other reported family VIII esterases, EH_7_ presents two different domains, a large α/β domain (residues 1–94 and 210–409) and a small helical domain (residues 95–209), constituted by a total of 11 α‐helices and 11 β‐sheets (Fig. 3A). The α/β domain is composed of a central β‐sheet with seven antiparallel β‐strands (β1, β2, β7, β8, β9, β10, β11) surrounded by two α‐helices at one side (α1, α11) and four α‐helices at the other (α2, α7, α9, α10). There is an extra motif on top of the central β‐sheet composed of one additional α‐helix (α8) and two antiparallel extra β‐strands (β5, β6). The small helical domain involves four α helices (α3, α4, α5, α6) and two antiparallel β‐strands (β3, β4) (Fig. 3A). The large active site cavity, which can be split into two parts, S1 and S2, is located at the domain interface (Fig. 3B) and is outlined by three prominent regions that may regulate the entrance/leaving of the substrates and make the bulk of the binding sites. S1 is bordered by regions R1 (Phe230‐Gly275) and R2 (His139‐Thr169) from each domain, while S2 is defined by β9 at the ground and R3 (Met312‐Gly336), all at the α/β domain. The catalytic Ser76 is covered by Phe147 (from R2) and Met366 (from the β9‐β10 turn), which configure a tunnel (Fig. 3B inset), giving access to the catalytic pocket. There are two cis peptides, His119‐Pro120 located at the β3‐β4 turn in the small helical domain and Ile388‐Pro389 at the β11‐α11 turn in the α/β domain.

Analysis of EH_7_ folding using the *DALI* [21] server was employed to search for homologous proteins. The closest homologues are two esterases isolated from environmental samples, *i.e*. EstU1 with 37% identity and RMSD of 1.9 Å on 376 Cα atoms (PDB code 4IVI) [11], Est‐Y29 with 30% identity and RMSD of 2.1 Å on 373 Cα atoms (PDB code 4P6B) [22] and EstB from *B. gladioli* with 32% identity and RMSD of 2.0 Å on 351 Cα atoms (PDB code 1CI8) [23]. All these proteins belong to family VIII of the carboxylesterases and show very similar α/β and small helical domains. However, major differences were observed in the R2 and R3 regions, as shown in Fig. 3C. First, Est‐Y29 and EstB present a much longer R1, which in EstB is very close to the catalytic site, resulting in more restricted access for substrates; this possibly explains the absence of β‐lactamase activity reported in this enzyme. Remarkably, the R1 region of EH_7_ is clearly retracted from the active site, leaving a wide and open cavity at the S1 site that, therefore, may allocate a wide spectrum of substrates without any steric hindrance and providing a possible explanation for the high promiscuity of this enzyme. On the other hand, the high B‐factor values observed at the R2 and R3 segments (Fig. 3D) suggest that both are highly flexible and must have an essential role in fitting the substrates into the active site cavity.

### EH_7_ makes an octameric assembly

**Fig. 4:**
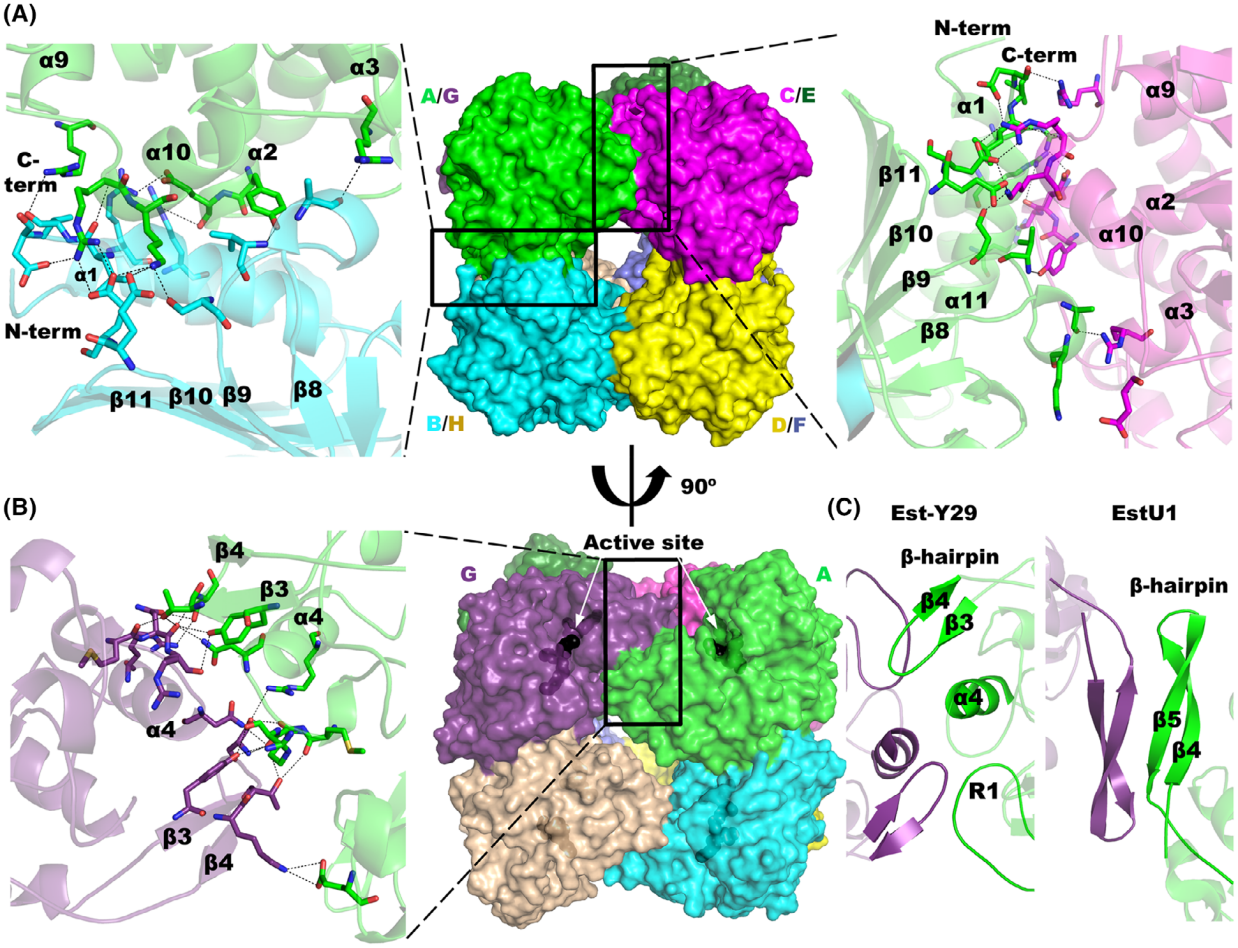
The EH_7_ octamer. (A) View along the fourfold axis showing the interaction between assemblies AB (left, green‐blue) and CA (right, magenta‐green). (B) A right‐handed 90° rotation showing the weaker AG interface that is formed through the corresponding small helical domains (green‐violet). The EH_7_‐O‐4NHP found in the complex is displayed as black spheres in the active sites of A and G. Hydrogen bonds and salt bridges are shown as dashed lines. (C) Interactions of the AG interface in the Est‐Y29‐F125W (PDB code 5ZWV) octamer are performed between β‐hairpin (β3‐β4), α4 and R1, while those in the EstU1 octamer are performed exclusively between the β‐hairpin (β4‐β5). Subunits A and G are shown as cartoons coloured green and violet, respectively. All these figures were depicted using the software pymol (PyMOL Molecular Graphics System, Schrödinger LLC).

EH_7_ is presented as a biological homo‐octamer with approximate dimensions of 8.9 × 8.6 × 7.5 nm, which is assembled in a 422‐point symmetry arrangement (Fig. 4A,B) that buries 18% of its total surface area. The asymmetric unit of the wild‐type crystal contains a tetramer that generates the octamer by binary crystallographic symmetry, while the whole octamer is formed by noncrystallographic symmetry in the complexes. The subunits interact through two different types of interfaces, AB and AG (Tables S1 and S2). In the interface type AB, many polar interactions take place between secondary structure motifs of the α/β domain (Fig. 4A). Hydrogen bonds mainly involve α1, α2, α3, α10, the loops connecting α9‐α10 and β8‐β9, and the C‐terminus. Salt bridges of the AB interface involve motifs α1, α2 and α10, the loop connecting β10‐β11 and the C‐terminus. On the other hand, interface AG (Fig. 4B) seems weaker and displays fewer interactions that are among residues of the small helical domains, *i.e*. hydrogen bonds within α4, β3 and loops connecting β3‐β4 and α3‐α4, whereas salt bridges are made between β3 and the loop connecting α10‐β7. This AG‐type pair allocates their corresponding active sites in the exterior of the octamer (Fig. 4B), located 40 Å apart.

Among all EH_7_ homologues, Est‐Y29 is reported to form a homo‐octamer as the biological assembly. In addition to the interaction through the β‐hairpin (β3‐β4) and α4 described in the EH_7_ AG interface, the larger R1 of Est‐Y29 also interacts with the β‐hairpin of the adjacent subunit, making a more compact octamer (Fig. 4C). Interestingly, EstU1 makes a similar tetrameric assembly to that formed by EH_7_ and Est‐Y29, but the two tetramers interact exclusively through the longer β‐hairpins from each subunit (Fig. 4C). As a consequence, the second tetramer moves away almost 20 Å, making a weaker interface, but turns around by 40° in a way that each pair of active sites is at 45 Å, which is an equivalent distance to that found in EH_7_ and Est‐Y29. On the other hand, EstB forms a different association into a dimer with both subunits arranged through the extension of their central β‐sheet, similar to that observed in other esterases. In fact, it does not present the β‐hairpin that seems to be a key feature involved in octamer formation. Thus, EH_7_, EstU1 and Est‐Y29, all of them isolated from soil samples, make equivalent molecular assemblies that may reflect a common biological role in nature, and the differences observed in R1, mostly in the β‐hairpin, described above (Fig. 3C) might be fine‐tuning their specificity in their particular environment.

### EH_7_ presents an open active site

**Fig. 5:**
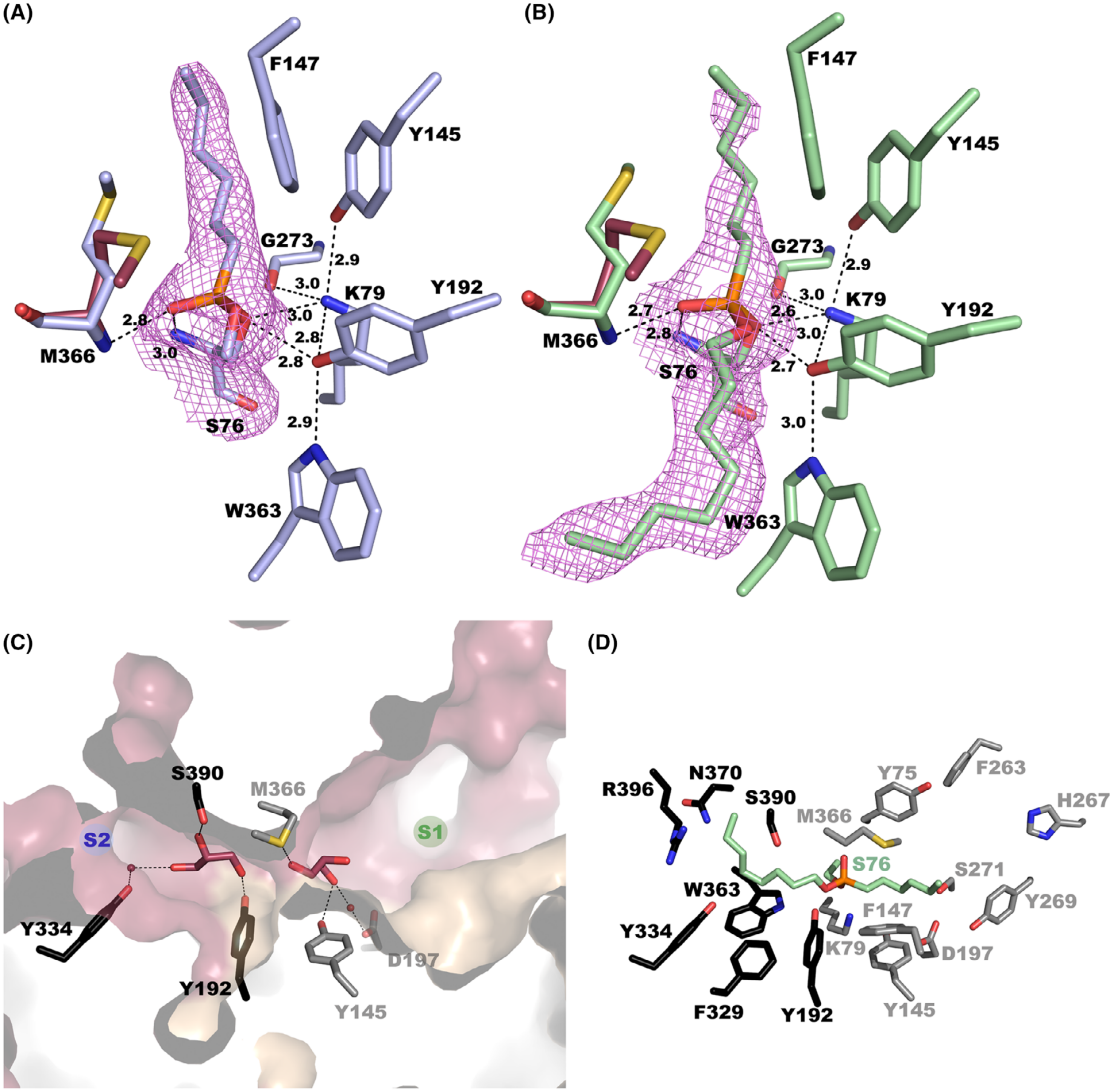
Active sites of EH_7_ complexed with M‐4NHP (A, blue) and with O‐4NHP (green, B). Hydrogen bond networks are shown as dashed lines for M‐4NHP and O‐4NHP. Met366 for the wild‐type protein is shown as raspberry sticks. 2Fo‐2Fc electron maps contoured at 1.0 σ for M‐4NHP and O‐4NHP (violet). (C) A cross‐section of the molecular surface of EH_7_ showing the catalytic pocket in the substrate‐free wild‐type enzyme. The surface is coloured following the pattern of Fig. 3B, the small helical domain in wheat and the α/β domain in prune. (D) A view of the phosphonate inhibitor complex, with residues bordering the alcohol (black)‐ and acyl (grey)‐ binding sites. All these figures were depicted using the software pymol (PyMOL Molecular Graphics System, Schrödinger LLC).

Soaking experiments of EH_7_ crystals into a solution containing either EH_7_‐M‐4NHP or EH_7_‐O‐4NHP were undertaken to depict the active site. Ser76 performs a nucleophilic attack on the phosphonate moiety of these substrates, releasing nitrophenyl and resulting in a covalent complex that mimics the tetrahedral intermediate of the first acylation step in the hydrolytic reaction (Fig. 5A,B). The catalytic triad of EH_7_ is formed by Ser76, Lys79 and Tyr192. Although the role of Tyr and Lys has been a matter of controversy, a recent study has given experimental evidence to propose that Tyr might act as a general base, whereas Lys could be the general acid aiding the positioning and polarization state of Tyr [9]. Nucleophilic serine and lysine are found in the S‐X‐X‐K motif, located at the beginning of α2, while conserved Tyr192 is located before α6. The resulting phenolate of Tyr192 is stabilized by the side chains of Trp363 (at β9) and Lys79, the last residue being hydrogen‐bonded to Tyr145 (R2) and Gly273 from the ^272^G‐G‐X‐G^275^ motif (at the end of R1). This net of polar links contributes to fixing a constrained geometry of the catalytic site in the intermediate (Fig. 5A,B). In addition, the oxoanion hole stabilizing the negatively charged oxygen is formed by Ser76 and Met366 backbone amides. Recently, three different subfamilies differing in some sequence motifs have been described within family VIII esterases [10]. According to this classification, EH_7_ belongs to subfamily VIII.1 (Table 1). Trp363 within the W‐X‐G motif, Tyr145 and the G‐G‐X‐G motif are all conserved among subfamilies VIII.1 and VIII.2 but not in subfamily VIII.3, class C β‐lactamases or transpeptidases, highlighting potential molecular mechanism differences within this superfamily of proteins.

Structural superimposition of the wild‐type subunit onto those from the complexed structures shows no significant structural changes in the EH_7_ active site upon complex formation. The only difference observed in the complexes is a rearrangement of the Met366 side chain (Fig. 5A,B) that, together with Phe147, makes a ceiling over Ser76 (Fig. 3C), making a narrow path to access the catalytic site that probably must retract to allow substrate entrance. While methionine is nonconserved, Phe is common to enzymes from subfamily VIII.1. Interestingly, mutagenesis of Phe147Ala did not influence the substrate specificity of the hydrolase, as this mutant was able to hydrolyse the same number of substrates, albeit most of them (93% of the esters tested) at lower rates (Fig. 2A), which points to a putative crucial role of Phe as a gate giving access to the catalytic site and possibly to the positioning of the substrates during catalysis. The flexibility of the R2 and R3 regions, as revealed by their high B‐factor values mentioned above, suggests that other structural changes might also occur that allow for the binding of other bulky substrates into the active site.

Figure 5 gives a detailed picture of the residues delineating the active site in the wild‐type and EH_7_‐M‐4NHP and EH_7_‐O‐4NHP complexes. Structural comparison (not shown) of our complex with other reported EH_7_ family enzymes complexed with substrates, such as the Est‐Y29/(*S*)‐ketoprofen (PDB Code 5ZWR) [24] or the PcEst S57A/lovastatin (PDB Code 6KJD) [9] complexes, ascribes the wide S1 to the acyl‐binding site of EH_7_ and its homologues. As observed in Figs 5C,D and S1 site is an open cavity where apart from Tyr145 hydrogen‐bonded to Lys79, the side chains of Tyr75, Phe147 and Met366 create a hydrophobic environment for the proximal moiety of the acyl site, the remaining is exposed to the solvent due to its shorter and retracted R1. In the case of the unliganded crystal, a glycerol molecule has been trapped at S1, being involved in hydrogen bridges to Tyr145, Met366 and Asp197, the last through a water molecule, which means that some additional polar interactions may be formed upon substrate binding (Fig. 5C). On the other hand, S2, assigned to the alcohol‐binding site, is shaped by both hydrophobic and polar residues. The proximal C8 moiety of the inhibitor is located in a hydrophobic environment made by Phe329 and Trp363 (Fig. 5D) and extends towards one of the two channels shown in Fig. 5C, both delineated mostly by polar residues. A glycerol molecule has been trapped in this S2 site in the free enzyme, making hydrogen bonds with Tyr192, Ser390 and Tyr334, this last one through a water molecule (Fig. 5C). Furthermore, many water molecules are included in S2, meaning that S2 seems to have a mostly polar nature. Interestingly, mutagenesis of Tyr334Ser did not influence the substrate range but did influence the conversion rates (Fig. 2A); thus, among the 43 esters tested, 14 were hydrolysed at higher rates (from 1.8‐ to 7.5‐fold), whereas 7 were hydrolysed at lower rates (from 2.1‐ to 15.7‐fold). The ester whose hydrolysis rate was most promoted (ethyl (*R*)‐(+)‐4‐chloro‐3‐hydroxybutyrate; 7.53‐fold) corresponds to an ester with a logP value of 0.33 ± 0.26, whereas that whose rate was most attenuated (vinyl benzoate; 15.6‐fold) was an ester with a logP value of 2.25 ± 0.54 (logP values reported in [4]). Together, this finding points to a putative crucial role of Tyr334 in the catalytic site and possibly in the positioning of molecules with different polar characteristics, likely defined by the presence or absence of a water molecule.

### Molecular basis behind the broad range activity of EH_7_

**Fig. 6:**
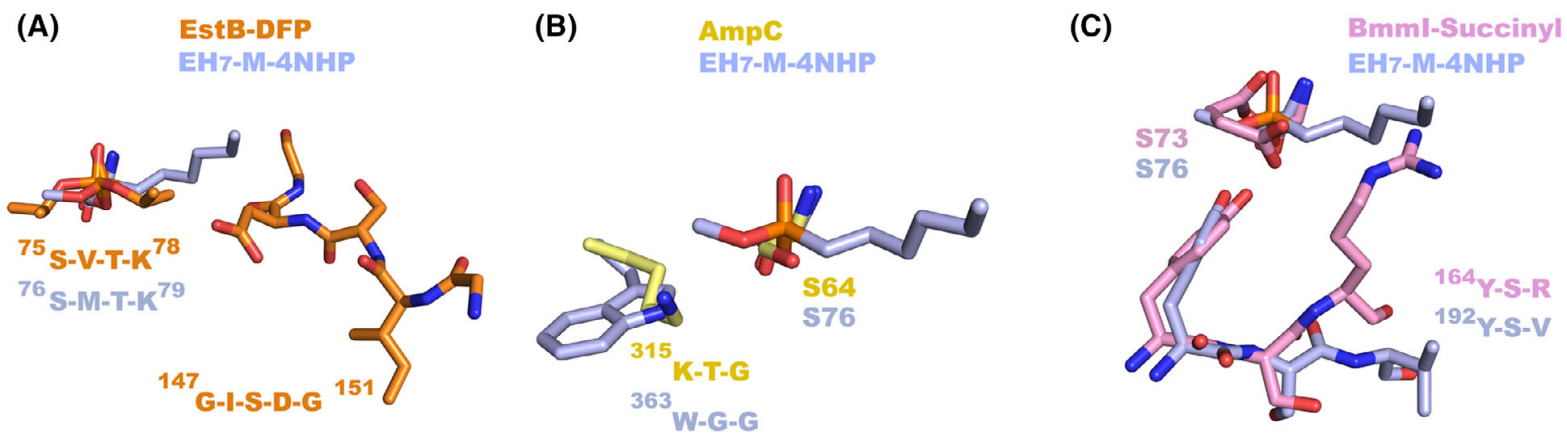
Conserved motives. (A) S‐X‐X‐K in EH_7_ complexed with M‐4NHP (blue) and EstB‐DFP (orange, PDB code 1CI9), and motif G‐X‐S‐X‐G shown by EstB‐DFP. (B) Motif K‐T‐G in AmpC (yellow, PDB code 1KE4) and W‐X‐G in EH_7_ complexed with M‐4NHP. (C) Motif Y‐X‐X in BmmI complexed with succinic acid (pink, PDB code 6KJT) and EH_7_ complexed with M‐4NHP (blue). All these figures were depicted using the software pymol (PyMOL Molecular Graphics System, Schrödinger LLC).

When comparing the active site of EH_7_ with its homologues, the G‐X‐S‐X‐G motif is absent in subfamilies VIII.1 and VIII.3 (Table 1). This motif is shared with family IV ester hydrolases that house the nucleophilic serine. In the case of EstB (a VIII.2 subfamily enzyme) (EstB‐DFP, PDB code 1CI9), this motif is within the R2 region that is very close to the active site, which could be the reason behind its lack of β‐lactamase activity [23]. EH_7_, lacking this motif, presents a more opened and exposed S1 site having no steric hindrance for the entrance of substrates to the active site (Fig. 6A), in agreement with its capacity to hydrolyse a broad range of substrates. Moreover, at the other wall of the S1 site, R1 is retracted from the active site, further enlarging the catalytic cavity of EH_7_, as previously mentioned (Fig. 3C), which may also explain the capacity to hydrolyse large substrates, such as nitrocefin (Fig. 2B), representing β‐lactamase activity.

On the other hand, the change of Lys to an aromatic residue at the β‐lactamase K‐T‐G box in the aminotranspeptidases has been recently proposed to determine acyltransferase activity by contributing to the proper allocation of aromatic acyl acceptors. According to this, the presence of Trp in subfamilies VIII.1 and VIII.2 (Table 1 and Fig. 6B) should suggest this activity within family VIII enzymes [25]. This hypothesis was verified by mutating H‐D‐G to W‐G‐G in EstA, a subfamily VIII.3 homologue, a change that transformed this hydrolase into an acyltransferase [25]. The acyltransferase activity of EH_7_ verified, as mentioned above, through the synthesis of benzyl 3‐oxobutanoate from benzyl alcohol and vinyl acetoacetate (Fig. 2B) reinforces this assumption.

However, a close inspection of the active site reveals that other structural determinants must be considered. Thus, the conserved motif Y‐X‐X containing the catalytic Tyr (Table 1) includes Ser193 and Val194 in EH_7_, two small residues enabling the entrance of large substrates. This motif is very close to the active site, and therefore, the residues with long side chains may generate steric hindrance with bulky substrates, as was observed in the lack of acylation capability of BmmI (PDB code 6KJT), which was attributed to the presence of Arg166 (Fig. 6C) [26]. Other EH_7_ homologues present larger residues at the Val194 position (Leu or His among others), which can be an additional molecular determinant behind the capacity of EH_7_ to hydrolyse bulky substrates, such as the mycotoxin T‐2 and the PET degradation product BHET. In conclusion, EH_7_ is a promiscuous ester hydrolase, probably due to its wide and opened catalytic cavity allowing the entrance of a wide spectrum of substrates, whose access and positioning may be influenced by Phe147 and Tyr334 (Table 1).

### Degradation of T‐2 mycotoxin and BHET by EH_7_

**Fig. 7:**
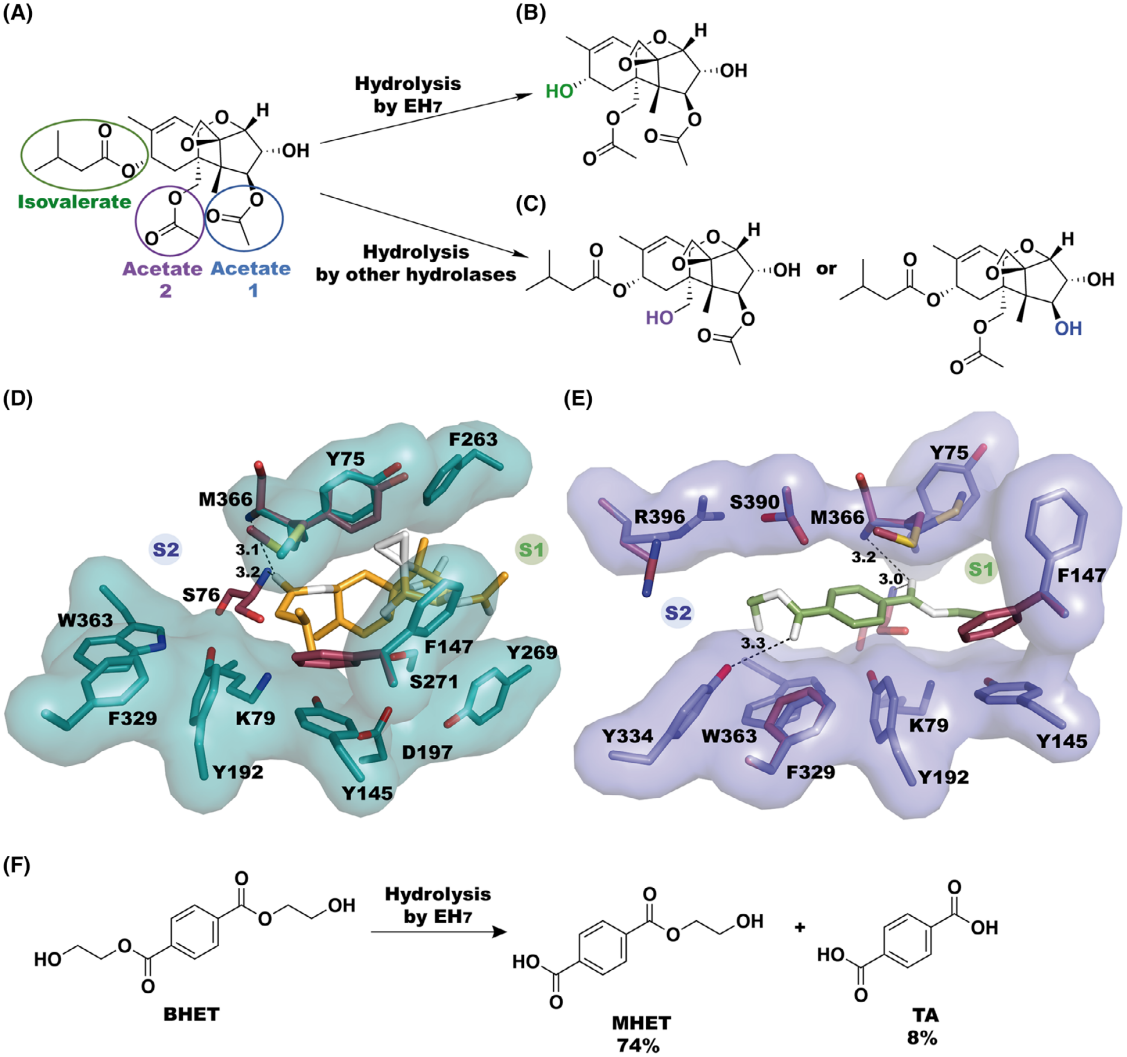
(A) Structure of toxin T‐2. Different ester bonds that can be hydrolysed are coloured. Product(s) obtained by the degradation of toxin T‐2 through the isovalerate group by EH_7_ (B) and by the hydrolysis of both acetates by different hydrolases (C). Depiction of the putative inversion of the acyl/alcohol‐binding modes shown by docking simulation with toxin T‐2 (D) and BHET (E). Residues considered flexible in the docking are shown in teal sticks with their surface shown in the same colour for T‐2 (D) and in slate for BHET (E). Unbound protein residues having a different conformation are shown as prune sticks. (F) Degradation of BHET performed by EH_7_, where MHET and TA are formed. Figs A, B, C and F were depicted using the software chemdraw (PerkinElmer Informatics). Figs D and E were depicted using the software pymol (PyMOL Molecular Graphics System, Schrödinger LLC).

T‐2 mycotoxin belongs to the trichothecenes toxin family, which is composed of a central six‐membered ring with an oxygen atom containing an epoxide functionality. This core is completed by two additional (six‐ and five‐membered) rings (Fig. 7A). Trichothecenes are divided into four categories, A, B, C and D, depending on the substitution pattern [27, 28], with T‐2 belonging to type A, which is the most toxic mycotoxin that includes immuno, neuro and reproductive toxicity [29]. Interestingly, EH_7_ is the only ester hydrolase that hydrolyses T‐2 at the isovalerate group (Fig. 7B), while the others remove the two acetates (Fig. 7C).

In an attempt to understand the structural basis behind the unique EH_7_ hydrolysis of T‐2, a docking simulation was performed to allocate the T‐2 toxin into the active site using the coordinates of free EH_7_. For the induced‐fit analysis, most residues that were close to the inhibitors in the complexes shown in Fig. 5D were considered flexible. Surprisingly, most solutions showed an inversion of the binding mode of T‐2 with respect to the acyl/alcohol sites observed in the EH_7_‐M‐4NHP and EH_7_‐O‐4NHP complexes. Indeed, the bulky alcohol moiety finds a better location at the wider and solvent‐exposed S1 cavity while positioning the small isovalerate moiety at S2. Among all results, the best solution was chosen on the basis of its productive interaction geometry with the catalytic triad (Fig. 7D), with the ester carbonyl carbon placed at an optimal distance to be attacked by Ser76 and its free oxygen making polar links to the NH from Ser76 and Met366. Interestingly, the docking model shows an almost identical conformation for most residues that were allowed to move in the simulation. The only exception is Phe147, which flips and retracts from the catalytic site to allow the correct position of the bulky toxin T‐2, apparently supporting its role as a phenyl gate modulating the entrance of substrates to the active site mentioned above. This essential role of the Phe side chain agrees with the decreased activity observed in the Phe147Ala mutant with respect to all substrates while maintaining a selectivity profile similar to that of native EH_7_ (Fig. 2A). On the other hand, the inversion in the acyl/alcohol‐binding sites suggested by the docking simulation with T‐2 would agree with the activity assays of mutant EH_7Y334S_, which shows a superior hydrolytic capacity *versus* substrates with large acyl groups than the native enzyme (Fig. 2A), pointing to Tyr334 being part of the acyl‐binding site.

As previously mentioned, EH_7_ converts BHET into MHET and TA (Fig. 7F). To disclose this activity, BHET was docked into the EH_7_ active site using the same simulation strategy as with T‐2. Interestingly, all solutions invert the acyl/alcohol‐binding sites similar to the trend observed with the bulkier T‐2 molecule and show the flipped conformation of Phe147. The best model, represented in Fig. 7E, shows that Met366 also presents a conformational change, as observed upon the formation of the EH_7_‐M‐4NHP and EH_7_‐O‐4NHP complexes, which nevertheless keeps the sulphur atom at a similar distance to the Phe147 aromatic ring of 5.5 Å that, in turn, is similar to the 5.0 Å found in the free enzyme. In the case of the T‐2 toxin, a reorganization of Met366 was also observed in the complex model, but the allocation of the bulkier alcohol moiety increased the Met366 to Phe147 distance up to 6.5 Å. In any case, it seems apparent that the combined positioning of the pair Met366/Phe147 must be crucial in EH_7_ activity, possibly supporting the proper geometrical integrity of the catalytic triad. Thus, the Phe147 side chain is only 4 Å from the catalytic Tyr192 in the free enzyme and the tetrahedral intermediate complexes, while a flip is required to allow the entrance of the substrate, as suggested by the docking simulation, or the product release. Furthermore, the model shows that the acyl moiety of BHET accommodates nicely at the S2 site (Fig. 7E), where it is stabilized through a hydrogen bond from Tyr334 to the ester‐free oxygen. The same binding mode may be attributed to the carboxylate moiety of the MHET intermediate to produce TA in the last step, which can explain the unique activity of EH_7_ *versus* BHET. This is consistent with the deleterious decrease in the hydrolytic activity of the EH_7Y334S_ mutant *versus* BHET (0.07 ± 0.01 units·g^−1^).

This switch in the acyl/alcohol‐binding sites has been previously described upon double mutation of the thermophilic esterase EST2 M211S/R215L, when complexed with 1‐hexadecanesulfonyl chloride [30]. At first, this double mutation was designed to allow for the binding of longer acyl substrates at the S2 site, but unexpectedly, the acyl moiety of the ligand was inverted *versus* previously reported complexes with the enzyme [31]. Furthermore, this duality of the acyl‐ and alcohol‐binding pockets was also reported on promiscuous human carboxyl esterase I (hCE1) depending on the chemical structure of the bound substrates [32]. Therefore, the same plasticity may operate in EH_7_, which may represent an additional molecular mechanism assisting its broad enzymatic specificity.

## Conclusions

In this work, we have carried out an extensive biochemical and structural characterization of EH_7_, an ester hydrolase belonging to family VIII, in particular subfamily VIII.1, with a broad and unusual substrate profile. In detail, EH_7_ is a substrate promiscuous enzyme with efficient hydrolase and acyltransferase activities. It is characterized by a wide and prominent catalytic cavity accepting and capable of hydrolysing an ample set of different esters, some of which are relevant to plastic and mycotoxin degradation, divided into two areas, S1 and S2. S1 is an exposed area and is composed of R1 and R2 regions delineating both walls of it. The retraction of these two regions from the catalytic triad of EH_7_ was found to play a critical role in its substrate ambiguity, which was also promoted by the presence of a conserved motif W‐X‐G shown by subfamily VIII.1.

## Materials and methods

### Source and purification of EH_7_

The vector pBXCH and the host *Escherichia coli* MC1061 were the sources of His6‐tagged EH_7_ (GenBank acc. nr. KY483644) [4], which was produced and purified at 4 °C after binding to a Ni‐NTA His‐Bind resin (from Merck Life Science S.L.U., Madrid, Spain) as described previously [33]. After the Ni‐NTA column, the protein was further purified by size‐exclusion chromatography using a HiLoad 16/60 Superdex 200 column equilibrated in buffer: 40 mm N‐(2‐hydroxyethyl)piperazine‐N′‐(2‐ethanesulfonic acid) (HEPES) pH 7, 50 mm NaCl, 1 mm dithiothreitol (DTT). Two peaks were obtained after purification and concentrated independently: the first peak from 53.5 to 57.5 mL (concentrated at 44 mg·mL^−1^) and the second peak from 64.5 to 66.5 mL (concentrated at 28 mg·L^−1^) were stored at 193 K. The purity of all the samples was confirmed by SDS/PAGE.

### Site‐directed mutagenesis

Mutagenic PCR was performed using the QuikChange Lightning Multi Site‐Directed Mutagenesis Kit (Agilent Technologies, Cheadle, UK), as described previously [34], and mutants were processed as the wild‐type protein. The forward primers used to generate the EH_7Phe147Ala_ and EH_7Tyr334Ser_ variants were as follows: 5′‐ TCAGGTCTGACCTATGGCGCCATGAACCGTACCAATGTG‐3′ and 5′‐ CAGCCTCGGAAACCCCGTCGGATGGCACGGGTTTTGGC‐3′, respectively.

### Enzyme characterization

The hydrolysis of carboxylic esters (the source and brand of each of the esters [purity ≥ 99%] used in this study was Merck Life Science S.L.U.), including BHET (ref. 465151) and T‐2 mycotoxin (ref. T4887) was assessed using a pH‐indicator assay in 384‐well plates (ref. 781162, Greiner Bio‐One GmbH, Kremsmünster, Austria) in a Synergy HT Multi‐Mode Microplate Reader. Reactions were followed in continuous mode at 550 nm (ε of phenol red, 8450 m ^−1^·cm^−1^) over 1–10 min, depending on the substrate [33]. Reaction conditions: (protein): 0.5 μg per well; (substrate): 20 mm (from a stock solution 200 mm in dimethylsulfoxide); T: 30 °C; pH, 8.0 (5 mm 4‐(2‐hydroxyethyl)‐1‐piperazinepropanesulfonic acid (EPPS) buffer, plus 0.45 Phenol Red®); reaction volume: 40 μL. These standard conditions were used to determine the kinetic parameters for carboxylic esters but using substrates and enzyme concentrations as reported previously [5]. Also, for the determination of the optimum temperature, but the model ester glyceryl tripropionate was used as the substrate (because of its higher stability at high temperatures compared with *p*‐NP propionate), and the temperature ranged from 20 to 60 °C (reaction time, 1 min). Reaction products for BHET were examined by HPLC, as previously reported [19]. To determine the optimum pH, the model ester *p*‐NP propionate was used. The activity was assessed in 96‐well plates (ref. 655801, Greiner Bio‐One GmbH) in 50 mm Britton and Robinson (BR) buffer at pH 3.0–10.0 and 30 °C by monitoring the production of 4‐nitrophenol at 348 nm (pH‐independent isosbestic point, ε = 4147 m ^−1^·cm^−1^) over 5 min and determining the absorbance per minute from the generated slopes [35]. Reaction conditions: (protein): 0.5 μg per well; (*p*‐NP propionate): 0.8 mm (from a 40 mm stock solution in acetone); pH: 50 mm Britton–Robinson (BR) buffer, pH ranging from 3 to 10; reaction volume: 200 μL; and T: 30 °C. The hydrolysis of the chromogenic β‐lactamase substrate nitrocefin (484400‐5MG from Merck Life Science S.L.U.) was followed at 486 nm. This substrate undergoes a distinctive colour change from yellow as the amide bond in the β‐lactam ring is hydrolysed by β‐lactamase. The reaction conditions were as follows: (EH_7_), 2 μg per assay; (nitrocefin), 0–200 μm (from a 5 mg·mL^−1^ stock solution in DMSO); reaction volume, 100 μL; T, 30 °C; pH, 7.0 (40 mm HEPES buffer); and reaction time, 20 min. Reactions were carried out in 96‐well plates (ref. 655801, Greiner Bio‐One GmbH). All values were determined in triplicate by determining the absorbance per minute from the generated slopes and were corrected for nonenzymatic transformation [33, 35].

Acyltransferase activity was monitored by following the formation of benzyl 3‐oxobutanoate from the reaction of benzyl alcohol (50 mm), as an acyl donor, with a fourfold excess of vinyl acetoacetate (200 mm), adapting a protocol described elsewhere [25]. Both substrates were provided by Merck Life Science S.L.U. Reactions were performed in aqueous buffer (100 μL), namely, 5 mm EPPS buffer, pH 8.0, and 30 °C. Reactions were carried out in 1.5 mL Eppendoff®. After 60 min of incubation, 0.9 mL dimethylsulfoxide was added, and the samples were analysed by HPLC. The formation of benzyl 3‐oxobutanoate was followed by HPLC performed using a quaternary pump (Model 600, Waters, Milford, MA, USA) coupled to an autosampler (Varian ProStar, Model 420, Palo Alto, CA, USA) and a Zorbax Eclipse Plus C‐18 column (4.6 × 100 mm, 3.5 μm, Agilent Technologies) at 40 °C. The injection volume was 20 μL. The mobile phase was acetonitrile/H_2_O degassed with helium and acidified with 0.1% (v/v) formic acid with a fixed flow rate of 1.0 mL·min^−1^. The gradient consisted of an initial 15% (v/v) acetonitrile to 85% (v/v) in 20 min, followed by an additional 10 min to re‐equilibrate the column under the initial conditions. The detection of peaks was performed using a photodiode array detector (ProStar, Varian), and quantification was carried out using the software Varian Star LC workstation 6.41 at a wavelength of 220 nm. A calibration curve with benzyl alcohol and benzyl 3‐oxobutanoate standards was made between 0 and 10 mm.

### Crystallization and X‐ray structure determination of EH_7_ and EH_7_ complexed with a derivative of methyl (EH_7_‐M‐4NHP) and octyl‐4‐nitrophenyl hexylphosphonate (EH_7_‐O‐4NHP)

Initial crystallization conditions were explored by high‐throughput techniques with a NanoDrop robot (Innovadyne Technologies Inc., Wilmington, DE, USA) using 12 or 22 mg·mL^−1^ protein concentrations in HEPES (40 mm, pH 7), NaCl (50 mm), different protein:reservoir ratios and the commercial screens SaltRx, Index (Hampton Research, Aliso Viejo, CA, USA), JBScreen PACT++, JBScreen JCSG++ and JBScreen Classic (Jena Bioscience, Jena, Germany). After some optimization steps, including the addition of different additives, the datasets collected impeded crystal structure determination. Then, size‐exclusion chromatography was added to the purification step, and optimization was carried out with samples from each chromatography peak. Bar‐shaped crystals of EH_7_ were grown after 4 days using samples from the first peak protein (1 μL, 12 mg·L^−1^) in HEPES (40 mm, pH 7), NaCl (50 mm), DTT (1 mm), NaI (0.2 μL from a stock of 1 m) and the precipitant solution (1 μL, 22% PEG PEG3350, 0.1 m Bis‐Tris propane pH 8.5, 0.2 m NaF). The complex with the suicide inhibitors methyl and octyl 4‐nitrophenylhexyl phosphonate (EH_7_‐M‐4NHP and EH_7_‐O‐4NHP, respectively) was obtained by soaking crystals of EH_7_, grown as above, in the precipitant solution supplemented with 10 mm of either M‐4NHP or O‐4NHP for 3 h. Both inhibitors (ID ESIC01 and ESIC03) were provided by EUCODIS Bioscience GmbH (Vienna, Austria).

For data collection, crystals were transferred to cryoprotectant solutions consisting of mother liquor and glycerol (20% [v/v]) before being cooled in liquid nitrogen. Diffraction data were collected using synchrotron radiation on the XALOC beamline at ALBA (Cerdanyola del Vallés, Spain). Diffraction images were processed with XDS [36] and merged using AIMLESS from the CCP4 package [37]. All crystals present P422 point symmetry, with four molecules in the asymmetric unit in the wild‐type, eight molecules in the asymmetric unit in both complexes, and 47–49% solvent content within the unit cell. The data collection statistics are given in Table S3. The structure of EH_7_ was solved by molecular replacement with MOLREP [38] using the coordinates from the homologue EstU1 as a template (PDB code 4IVK). The structure of the complex was solved by difference Fourier synthesis using the coordinates of the EH_7_ native crystals. Crystallographic refinement was performed using the program REFMAC [39] within the CCP4 suite with automatic local noncrystallographic symmetry (NCS). For the wild‐type, amplitude‐based twin refinement was applied. The free R‐factor was calculated using a subset of 5% randomly selected structure‐factor amplitudes that were excluded from automated refinement. At the later stages, ligands were manually built into the electron density maps with Coot8 [40], and water molecules were included in the model, which, combined with more rounds of restrained refinement, reached the R factors listed in Table S3. For M‐4NHP and O‐4NHP, which are not present in the Protein Data Bank, a model was built using MacPyMOLX11Hybrid (The PyMOL Molecular Graphics System, Version 2.0, Schrödinger, LLC, New York, NY, USA). The model was used to automatically generate coordinates and molecular topologies, with GRADE (http://grade.globalphasing.org) for M‐4NHP and eLBOW [41] for O‐4NHP, suitable for REFMAC refinement. The figures were generated with pymol. The crystallographic statistics of EH7 are listed in Table S3.

### Docking simulations

The crystal structure of EH_7_ wild‐type was used as a template for the docking experiments. All resolved water molecules and other heteroatoms were removed from the structure prior to protein preparation and subsequent docking simulations. The coordinates of toxin T‐2 were generated using SMILES within Coot8 [40]. autodock Tools was used to create a .pdbqt file that is a modified PDB file containing the coordinates of the protein and substrate and additional information such as the atom type. Substrate docking was performed using AutoDock Vina [42] with Tyr75, Lys79, Tyr145, Phe147, Tyr192, Asp197, Phe263, Tyr269, Ser271, Phe329, Trp363 and Met366 defined as flexible side chains. The docking space was visually defined in autodock Tools. The grid box around the catalytic triad with dimensions of 26 Å × 28 Å × 34 Å was used to cover the entire substrate‐binding site. In the case of BHET, the coordinates were taken from the PDB code C8X. In this case, the flexible residues were Tyr75, Lys79, Tyr145, Phe147, Tyr192, Phe329, Tyr334, Trp363, Met366, Ser390 and Arg396. The grid box around the catalytic triad with dimensions of 32 Å × 36 Å × 40 Å was used. Default parameters were defined during docking with exhaustiveness of 16. Calculations were performed using the Lamarckian Genetic Algorithm (LGA) method. Among the results, the final model was chosen on the basis of its productive interaction with the catalytic triad, and small changes were manually made to properly fit into the active site.

## Conflict of interest

The authors declare no conflict of interest.

## Author contributions

JS‐A, IC‐R and MF wrote the original manuscript All the authors have given the approval to the final version of the manuscript; I C‐R and JS‐A performed the crystallization and X‐ray structure determinations and analysis; CC and MF contributed to protein expression, purification and characterization; JR and MV contributed to T‐2 degradation analyses. JLG‐A and FJP performed HPLC analysis of the reaction products.

### Peer review

The peer review history for this article is available at https://publons.com/publon/10.1111/febs.16554.

## Supporting information


**Fig. S1.** Raw data: ESI‐MS analysis of T‐2 degrading products.
**Table S1.** The EH_7_ octamer interfaces as calculated by Pisa.
**Table S2.** Atomic interactions at the interface.
**Table S3.** Crystallographic statistics of EH_7_, EH_7_‐4MP and EH_7_‐4OP.

## Data Availability

The atomic coordinates and structure factors for the EH_7_, EH_7_‐M‐4NHP and EH_7_‐O‐4NHP structures have been deposited in the RCSB Protein Data Bank with accession codes 7PP3, 7PP8 and 7PU6.
