# Supplementary material for: Crystal structure of a family VIII β‐lactamase fold hydrolase reveals the molecular mechanism for its broad substrate scope
Source: FEBS J. 2022 Jun 27;289(21):6714–30. doi: 10.1111/febs.16554 (PMC9795927; doi:10.1111/febs.16554)
Supplement: Supplementary file 1 — Fig. S1. Raw data: ESI‐MS analysis of T‐2 degrading products. Table S1. The EH7 octamer interfaces as calculated by Pisa. Table S2. Atomic interactions at the interface. Table S3. Crystallographic statistics of EH7, EH7‐4MP and EH7‐4OP. [file FEBS-289-6714-s001.zip › febs16554-sup-0001-Supinfo.pdf]

FEBS PRESS science publishing by scientists

WILEY

The FEBS Journal

www.febsjournal.org

Crystal structure of a family VIII β-lactamase fold hydrolase reveals the molecular mechanism for its broad substrate scope

Isabel Cea-Rama, Cristina Coscolín, Jose L. Gonzalez-Alfonso, Jog Raj, Marko Vasiljević, Francisco J. Plou, Manuel Ferrer and Julia Sanz-Aparicio

DOI: 10.1111/febs.16554

Supporting information

Crystal structure of a family VIII β-lactamase fold hydrolase reveals the molecular mechanism for its broad substrate scope

Isabel Cea-Rama, Cristina Coscolín, Jose L. Gonzalez-Alfonso, Jog Raj, Marko Vasiljević, Francisco J. Plou, Manuel Ferrer, and Julia Sanz-Aparicio

Table of Contents

1. Figure S1……2
2. Table S1……3
3. Table S2……4
4. Table S3……5
5. References……6

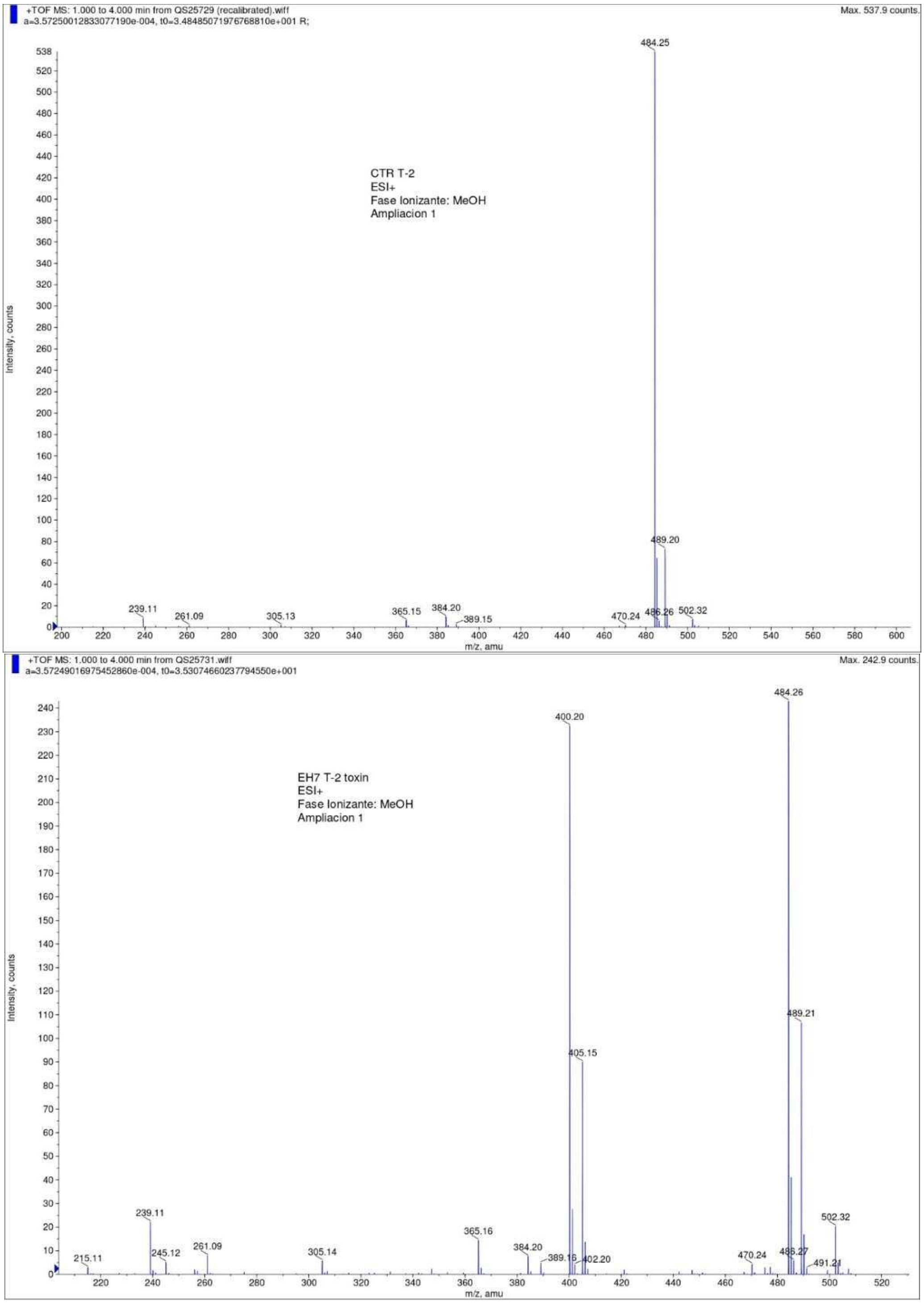

Figure S1. ESI-MS analysis of T-2 degrading products in the absence (upper) and in the presence (lower) of EH7. T-2 degradation assays were performed in 2 mL Eppendorf tubes with constant mixing in a shaker at 30 °C. A total of 2 µL of T-2 toxin stock solutions (from a stock solution of 100 mg/ml in acetonitrile) were added to 96 µL of 2 mM sodium bicarbonate buffer pH 7.0. Then, 2 µL of EH7 solution (from a stock solution of at least 10 mg/ml protein) was added. After 1 h, the reaction was stopped by adding 900 µL HPLC-grade methanol. The formation of degradation products was followed by mass spectrometry analyses. Conventional mass spectrometry analyses were performed on a hybrid quadrupole time-of-flight (QTOF) analyser, model QSTAR, Pulsar I, from AB Sciex (Framingham, MA, USA.). Reaction samples were analysed by direct infusion and ionized by electrospray ionization mass spectrometry (ESI–MS) with methanol as the mobile phase in positive reflector mode. High-resolution mass spectrometry (HR-MS) analysis was carried out by flow injection analysis combined with electrospray ionization mass spectrometry (FIA–ESI–MS) on a QTOF Agilent G6530A accurate mass QTOF liquid chromatography–mass spectrometry (LCMS) system (Agilent Technologies, Santa Clara, CA, USA.). The sample was directly infused and ionized by ESI in negative reflector mode. Ionization was enhanced by JetStream technology, and the mobile phase was 99.9:0.1 (v/v) H_2O/formic acid. Data were processed with Masshunter Data Acquisition B.05.01 and Masshunter Qualitative Analysis B.07.00 software (Agilent Technologies).

Tables

Table S1. **The EH_7 octamer interfaces as calculated by Pisa [43].**

**Interface**	**Area ($Å^2$)**	**$\Delta^i G$ (*kcal/mol*)**	**$\Delta^i G$ p (*value*)**	***N*$_{HB}$**	***N*$_{SB}$**	***N*$_{DS}$**
AB/DC/EF/HG/ CA/BD/GE/FH	1005.0	-3.8	0.49	6	6	0
AG/BH/CE/DF	388.7	2.7	0.85	16	2	0

Table S2. Atomic interactions at the interfaces.

	Hydrogen bonds			Salt bridges		
	**Molecule A**	**Dist. (Å)**	**Molecule B**	**Molecule A**	**Dist. (Å)**	**Molecule B**
AB	Tyr90(OH)	3.28	Val355(N)	Glu91(OE1)	3.08	Arg18(NH2)
	Glu91(O)	3.77	Arg21(NH1)	Lys305(NZ)	3.24	Glu376(OE1)
	Arg134(NH1)	3.15	Ala350(O)	Arg304(NH2)	3.16	Glu377(OE1)
	Lys305(NZ)	2.67	Ser357(OG)	Arg304(NH1)	2.73	Glu377(OE2)
	Arg304(N)	3.53	Leu407(O)	Lys305(NZ)	3.02	Glu377(OE2)
	Arg300(NH2)	3.49	Val408(O)	Arg304(NH2)	2.87	Asp409(OD2)
	**Hydrogen bonds**			**Salt bridges**		
	**Molecule A**	**Dist. (Å)**	**Molecule G**	**Molecule A**	**Dist. (Å)**	**Molecule G**
AG	Ser118(OG)	3.37	Arg158(NH1)	Glu331(OE1)	3.41	Lys115(NZ)
	Met148(O)	3.70	Thr116(OG1)	Glu331(OE2)	3.20	Lys115(NZ)
	Asn149(O)	2.92	Asn152(ND2)			
	Asn149(O)	3.76	Thr116(OG1)			
	Asn149(O)	3.55	Ser118(N)			
	Asn149(O)	3.35	Tyr114(OH)			
	Asn149(OD1)	2.81	Ser118(OG)			
	Arg150(O)	3.38	Tyr114(OH)			
	Arg158(NH1)	3.38	Ser118(OG)			
	Thr116(OG1)	3.65	Met148(O)			
	Tyr114(OH)	3.43	Asn149(O)			
	Ser118(N)	3.47	Asn149(O)			
	Asn152(ND2)	2.92	Asn149(O)			
	Thr116(OG1)	3.84	Asn149(O)			
	Ser118(OG)	2.90	Asn149(OD1)			
	Tyr114(OH)	3.35	Arg150(O)			

Table S3. Crystallographic statistics of EH_7, EH_7-4MP and EH_7-4OP.

Values in brackets are for the high-resolution shell			
Crystal data	EH7	EH7-M-4NHP	EH7-O-4NHP
Space group	P 43 2 2	P 43 21 2	P 43 21 2
Molecules/a.u.	4	8	8
Unit cell parameters			
a (Å)	102.94	150.64	149.54
b (Å)	102.94	150.64	149.54
c (Å)	331.82	325.26	321.72
Data collection			
Beamline	XALOC (ALBA)	XALOC (ALBA)	XALOC (ALBA)
Temperature (K)	100	100	100
Wavelength (Å)	0.97898	0.979240	0.979240
Resolution (Å)	49.04-2.25 (2.29-2.25)	49.63-2.65 (2.70-2.65)	48.77-2.92 (2.98-2.92)
Data processing			
Total reflections	596061 (31224)	577827 (28108)	504895 (29857)
Unique reflections	85057 (4503)	107165 (5270)	79916 (4498)
Multiplicity	7.0 (6.9)	5.4 (5.3)	6.3 (6.6)
Completeness (%)	99.5 (99.9)	98.4 (98.6)	99.9 (100.0)
Mean I/σ (I)	6.1 (2.3)	13.0 (2.5)	13.1 (2.8)
$R_{merge}^{\dagger}$ (%)	16.7 (69.6)	9.3 (69.0)	10.6 (66.9)
$R_{pim}^{\dagger\dagger}$ (%)	6.7 (27.6)	4.3 (32.6)	4.6 (28.1)
Molecules per ASU	4	8	8
Refinement			
R_{work} / $R_{free}^{\dagger\dagger\dagger}$ (%)	18.7/22.7	19.6/21.7	18.4/22.1
Nº of atoms/average B (Å^2)	13083/37.52	25363/48.65	25297/65.10
Macromolecule	12524/37.46	25128/48.71	25208/65.18
Ligands	75/59.46	43/68.72	6/71.15
Solvent	484/35.78	192/35.73	83/42.02
Ramachandran plot (%)			
Favoured	96.7	96.3	96.5
Outliers	0.06	0.3	0.3
RMS deviations			
Bonds (Å)	0.008	0.007	0.007
Angles (°)	1.414	1.468	1.446
PDB accession codes	7PP3	7PP8	7PU6

†Rmerge = ∑hkl ∑i | Ii(hkl) – (I(hkl))| / ∑hkl ∑i Ii(hkl), where Ii(hkl) is the ith measurement of reflection hkl and (I(hkl)) is the weighted mean of all measurements.

††Rpim = ∑hkl (1/(N - 1)) 1/2 ∑i | Ii(hkl) – (I(hkl))| / ∑hkl ∑i Ii(hkl), where N is the redundancy for the hkl reflection.

†††Rwork / Rfree = ∑hkl | Fo – Fc | / ∑hkl | Fo |, where Fc is the calculated and Fo is the observed structure factor amplitude of reflection hkl for the working / free (5%) set, respectively.

References

43 Krissinel E & Henrick K (2007) Inference of macromolecular assemblies from crystalline state. *J Mol Biol* **372**, 774–797.
